# Corporate Response Against COVID-19: Manufacturing Shift by Ford-Otosan

**DOI:** 10.5334/aogh.3434

**Published:** 2021-07-07

**Authors:** Anil Yasin Ar, Asad Abbas

**Affiliations:** 1 School of International Business and Logistics, Tecnologico de Monterrey, Queretaro, Mexico; 2 Writing Lab, Institute for the Future of Education, Tecnologico de Monterrey, Monterrey, Mexico

## Abstract

In the time of disasters and crisis, governmental organizations are responsible for performing disaster-relief tasks and providing economic support. However, these organizations may fail to fulfill their duties in emerging and developing market settings either because of a lack of resources or bureaucratic inefficiencies. At the rise of these situations, corporations are expected to help society via corporate social responsibility (CSR) projects. This viewpoint article reflects upon the Ford-Otosan’s attempt to devise a CSR project to support Turkish citizens during the COVID-19 crisis and meet the public’s expectations. This study aims to set a future research agenda on CSR and multinational joint-ventures’ non-market strategies by illustrating a unique insight into an international joint-venture during the COVID-19 crisis.

## Introduction

In the time of disasters and emergencies, governmental entities are tasked with the crisis management role. Governments have to cope with issues that arise from the social and economic perspectives of their countries. These efforts require a substantial number of resources, expertise, and workforce when it is a large-scale and international emergency response [1,2]. Even though governments should have already developed an agenda and designate funds to mitigate negativities during and aftermath of a disaster, in some cases, they may not have required resources readily available to aid their nations [3]. The lack of resources and inability to provide an instant response to disasters could be explained by a delay or inefficiencies in the socio-economic and socio-institutional networks [4]. In the dawn of these failures, enterprises could step in and support the governments and provide aids to societies through their own networks. These types of initiatives can help corporations in terms of boosting their corporate image, effectively mitigating their riskiness and exploiting non-market strategy building opportunities [5].

The forms of corporations’ support strategies vary. How they act and what range of response strategies that they build highly depends on the country settings that they are in and their organizational structure. This especially is the case for multinational corporations. They try to strategize ways of sustaining their businesses while supporting the host county communities [6]. While aiding a society during the crisis can be cumbersome for domestic corporations, it is even heftier task for the case for international joint ventures (IJVs). Not only do they have to solve their home base problems, but they also need to act together with their partnering firms to tackle host country struggles, such as “sustainable development, social and environmental resilience [7].”

COVID-19 has allowed scholars an opportunity to witness how the mechanics mentioned above work. Especially in emerging market settings, due to fragile economic conditions and socio-economic struggles, many firms are forced to actively participate actively in socio-economic relief projects. This viewpoint article, building upon the above discussion, provide an example through the case of Ford-Otosan’s (an international Joint Venture) corporate social responsibility (CSR) approach in Turkey. The reason Ford-Otosan provides a unique case is due to the firm’s ability to shift tis manufacturing process due to the COVID-19 as a CSR strategy and being the only Lighthouse-appointed (a designation only given to manufacturing sites that are leading the fourth industrial revolution) car manufacturing plant by World Economic Forum in the Middle East [8].

## The Case: Ford-Otosan at the Time of Covid-19

Ford-Otosan is one of the oldest car manufacturing IJVs, which was formed by Ford (U.S.A) and Otosan (Turkey) as a joint effort in 1959, based in Turkey. The corporation has four manufacturing sites and a distribution center in the country. The IJV is well-known for being the most prominent commercial vehicle production base in Europe with 44.000 units and famed as “the only Ford Plant in the “Lighthouse Factory” network by World Economic Forum [9].”

Regardless of its sheer size, Ford-Otosan is no exception to corporations that are adversely affected by the COVID-19. The IJV has been experiencing supply chain disruptions and production halts. For instance, in April 2020, Ford-Otosan declared that “…within the framework of the measures taken to reduce the effects of Coronavirus affecting the whole world, it was announced to the public that all our plants would be closed until April 27, 2020 [10].” This closure put the manufacturer behind the scheduled production plans and pre-determined deliveries.

However, when the corporation opened its doors to production in May 2020, instead of allocating all its resources to catch up with yearly manufacturing goals, Ford-Otosan CEO made the following announcement.

“At this point, I want to announce that we have started to manufacture components and prototypes with 3D (three-dimensional) printers and simple designs. The face masks we have designed are ready for production to help aid our healthcare professionals in the fight of COVID-19 [11].”

Taking action in a short period of time, the corporation managed to change its production lines from car manufacturing to medical equipment production. The IJV produced face mask, visor, and aerosol box prototypes for the health workers who are at the front line in Turkey’s fight against coronavirus. It illustrated that the corporation genuinely cares about the pandemic and its adverse social costs. Hence, it puts the CSR project priority above its profit-generation concerns.

Initially, the firm produced 30,000 visors allocated to 150 hospitals until the end of 2020 [12]. This first batch of visors’ designs was based on an open-source scheme. Hence, the corporation’s CSR strategy focused on manufacturing these essential items. However, Ford-Otosan realized the need to redesign the visors based on medical staff’s feedback. This marks the second stage. Not only did the firm allocate its manufacturing resources to this project, but it also dedicated its know-how and research and development (R&D) assets to the initiative. As a result, unlike the previous manufacturing methods, Ford-Otosan developed a technology that enabled the car manufacturer to produce visors exclusively from polyethylene terephthalate thermoplastic (PET) or polyethylene terephthalate glycol (PETG) polymer. This increased the health care providers’ health safety via covering their entire face at a 150-degree angle and access to high quality medical safety equipment that is made out of a high-grade polymer. As a third iteration, the IJV abandoned the hepa-seeded plastic masks, PET, and PETG used visors. Instead, a new prototype was developed with a commercial 3D printer. Not only does this iteration improved the mask’s sturdiness and comfort of use, but it also enabled Ford-Otosan to produce them in a more efficient manner. Hence, it managed to supply these to more than 150 hospitals all around Turkey at a consistent rate.

In the beginning of the 2021, the corporation reached a new milestone. Besides the aforementioned medical equipment, working with Republic of Turkish Health Ministry, it got the permission to start to produce face masks. Supply of this well-needed and difficult-to-find medical necessity tremendously helped the fight against COVID-19. As a result of these initiatives, Ford-Otosan attracted positive attention among citizens and international organizations. For instance, in a recent World Economic Forum publication, Ford-Otosan get an international recognition due to its free of charge critical medical supply donations to hospitals [13]. Also, all major domestic news outlets show big interest in the IJV’s CSR projects to prevent COVID-19. This gives a rise to positive public image for the corporation and reduction of liability of foreignness.

## Conclusion and Policy Recommendations

The case of Ford-Otosan provides a unique example of how the IJV reacts against COVID-19 in the host country context. It provides insight into the extent that a corporation would go to execute a CSR project in the event of an unprecedented catastrophe. It reveals how shifting a manufacturing strategy by an IJV in various iterations ensures the genuine solution to a significant social problem. Another important conclusion this case draws is the relationship between dynamic capabilities and CSR. This study shows that firms do not only rely on already existing skillsets to execute CSR projects. They dynamically seek to build new skills in order to adopt societies’ ever-changing needs and external market conditions. In addition, the Ford-Otosan case demonstrates that firms can attain public legitimacy and increase their firm reputation in domestic and international business chambers.

## Abbreviations

CSR: Corporate Social Responsibility

COVID-19: Coronavirus Disease 2019

IJV: International Joint Ventures

WEF: World Economic Forum

R&D: Research and Development

PET: Polyethylene terephthalate thermoplastic polymer

PETG: Polyethylene terephthalate glycol thermoplastic polymer

## References

[1] Tindall K, Hart P. Evaluating government performance during consular emergencies: Toward an analytical framework. Policy and Society. 2011; 30(2): 137–149. DOI: 10.1016/j.polsoc.2011.03.001

[2] Chien L-C, Lin R-T. COVID-19 outbreak, mitigation, and governance in high prevalent countries. Annals of Global Health. 2020; 86(1): 119–128. DOI: 10.5334/aogh.3011 32983915 PMC7500241

[3] O’Donovan K. Disaster recovery service delivery: Toward a theory of simultaneous government and voluntary sector failures. Administration & Society. 2019; 51(1): 120–139. DOI: 10.1177/0095399715622231

[4] Gonah L. Key considerations for successful risk communication and community engagement (RCCE) programmes during COVID-19 pandemic and other public health emergencies. Annals of Global Health. 2020; 86(1): 146–149. DOI: 10.5334/aogh.3119 33262935 PMC7678561

[5] Ar AY. Corporate social responsibility. In The Palgrave Encyclopedia of Interest Groups, Lobbying and Public Affairs. 2021; Cham: Palgrave Macmillan. DOI: 10.1007/978-3-030-13895-0_193-1

[6] Ar AY, Abbas A. Corporate social responsibility projects to supports multinational enterprises’ reputation building efforts in Mexico. Journal of Public Affairs. 2020; e2495. DOI: 10.1002/pa.2495

[7] Bloomfield C. Putting sustainable development into practice: Hospital food procurement in Wales. Regional Studies, Regional Science. 2015; 2(1): 552–558. DOI: 10.1080/21681376.2015.1100094

[8] World Economic Forum. Fourth industrial revolution: Beacons of technology and innovation in manufacturing. http://www3.weforum.org/docs/WEF_4IR_Beacons_of_Technology_and_Innovation_in_Manufacturing_report_2019.pdf. Accessed August 27, 2020.

[9] Ford-Otosan. Golcuk plant – Lighthouse factory with its industry 4.0 focused activities. https://www.fordotosan.com.tr/en/operations/production/plants. Accessed September 7, 2020.

[10] Public Disclosure Platform. Ford Otomotiv Sanayi A.Ş. Partial or complete suspension or impossibility of operations. https://www.kap.org.tr/en/Bildirim/839363. Accessed September 1, 2020.

[11] Daily Sabah. Ford Otosan to start producing masks in response to pandemic. https://www.dailysabah.com/business/ford-otosan-to-start-producing-masks-in-response-to-pandemic/news. Accessed October 11, 2020.

[12] Ford-Otosan. Support to health workers’ fight against coronavirus. https://www.fordotosan.com.tr/tr/medya/basin-kitleri/saglik-calisanlarinin-koronavirusle-mucadelesine-ford-otosandan-destek. Accessed September 7, 2020.

[13] World Economic Forum. How are companies responding to the coronavirus crisis? (2020). https://www.weforum.org/agenda/2020/03/how-are-companies-responding-to-the-coronavirus-crisis-d15bed6137. Accessed August 29, 2020.

